# Supplementary figures and images for: ZanzaMapp: A Scalable Citizen Science Tool to Monitor Perception of Mosquito Abundance and Nuisance in Italy and Beyond
Source: Int J Environ Res Public Health. 2020 Oct 27;17(21):7872. doi: 10.3390/ijerph17217872 (PMC7672598; doi:10.3390/ijerph17217872)

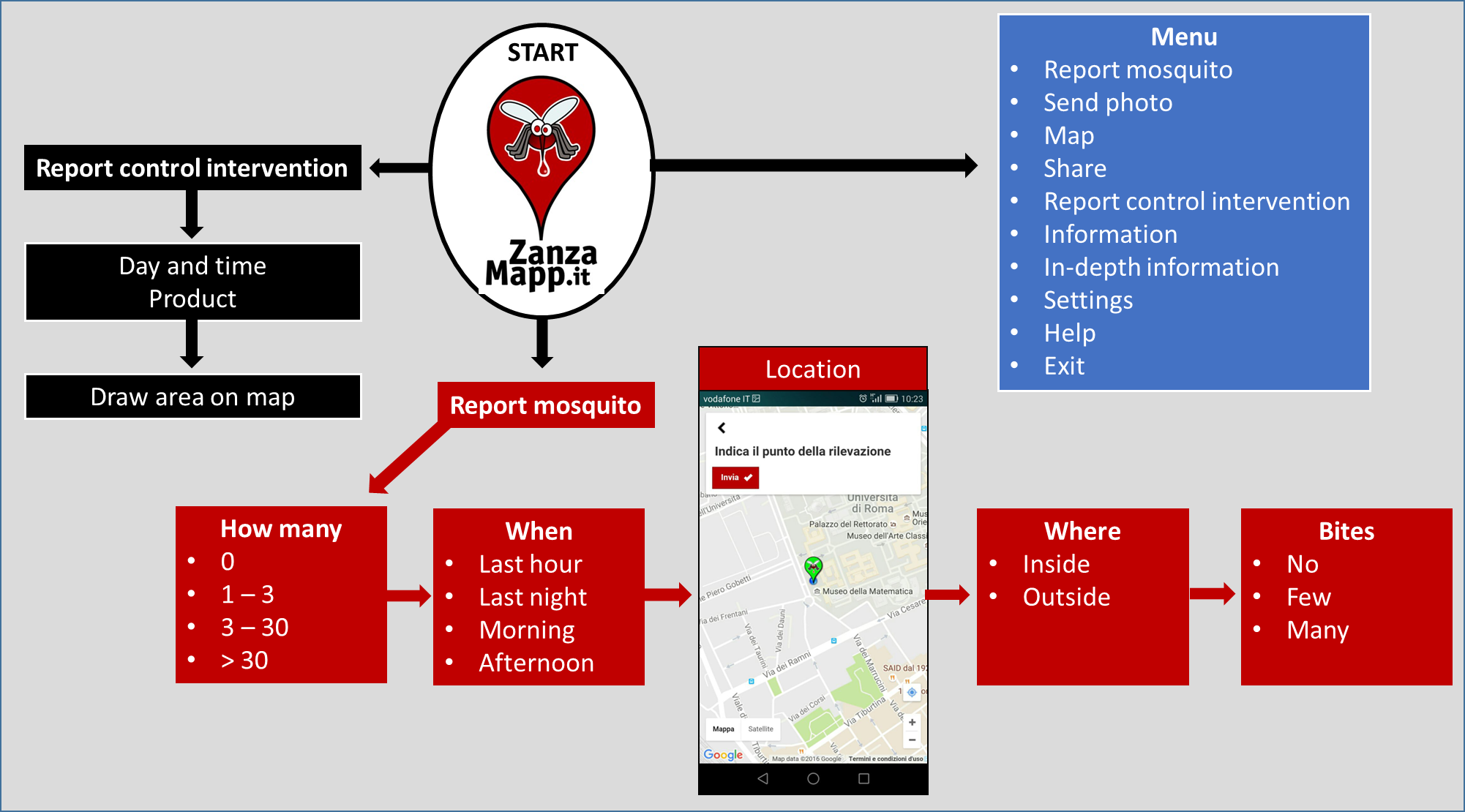

Supplement: Supplementary file 1 [file ijerph-17-07872-s001.zip › supmat/FigureS1.tiff]

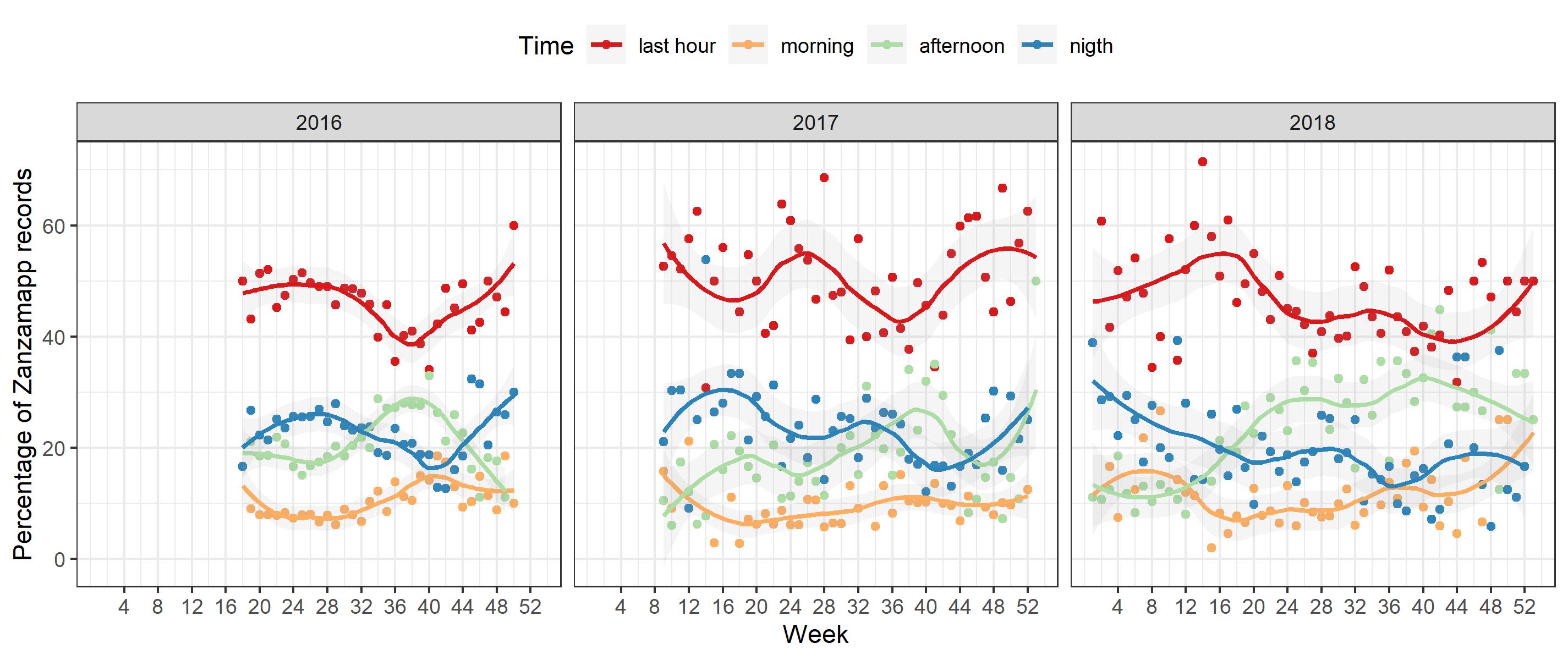

Supplement: Supplementary file 1 [file ijerph-17-07872-s001.zip › supmat/FigureS2.jpeg]

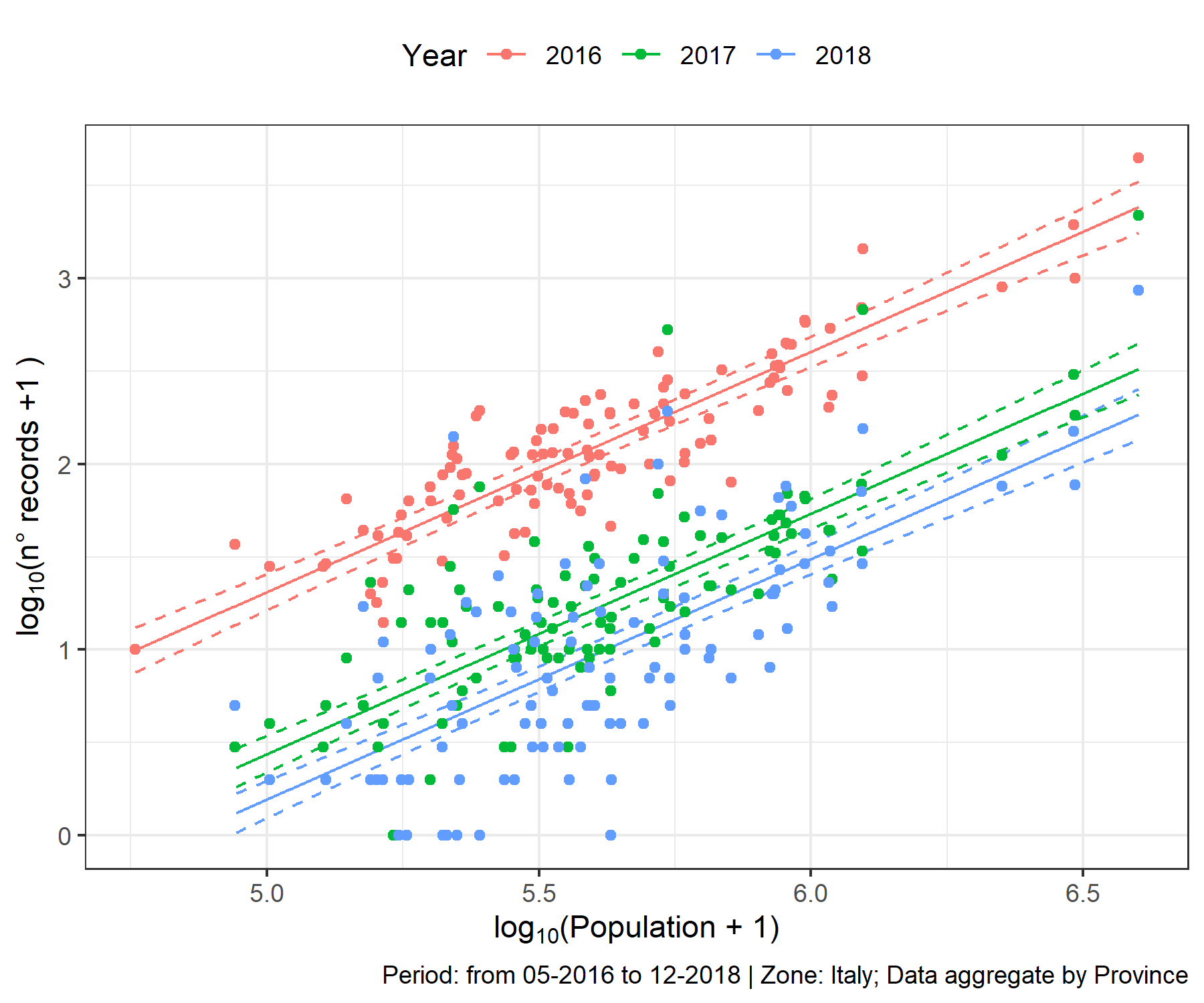

Supplement: Supplementary file 1 [file ijerph-17-07872-s001.zip › supmat/FigureS3.tiff]

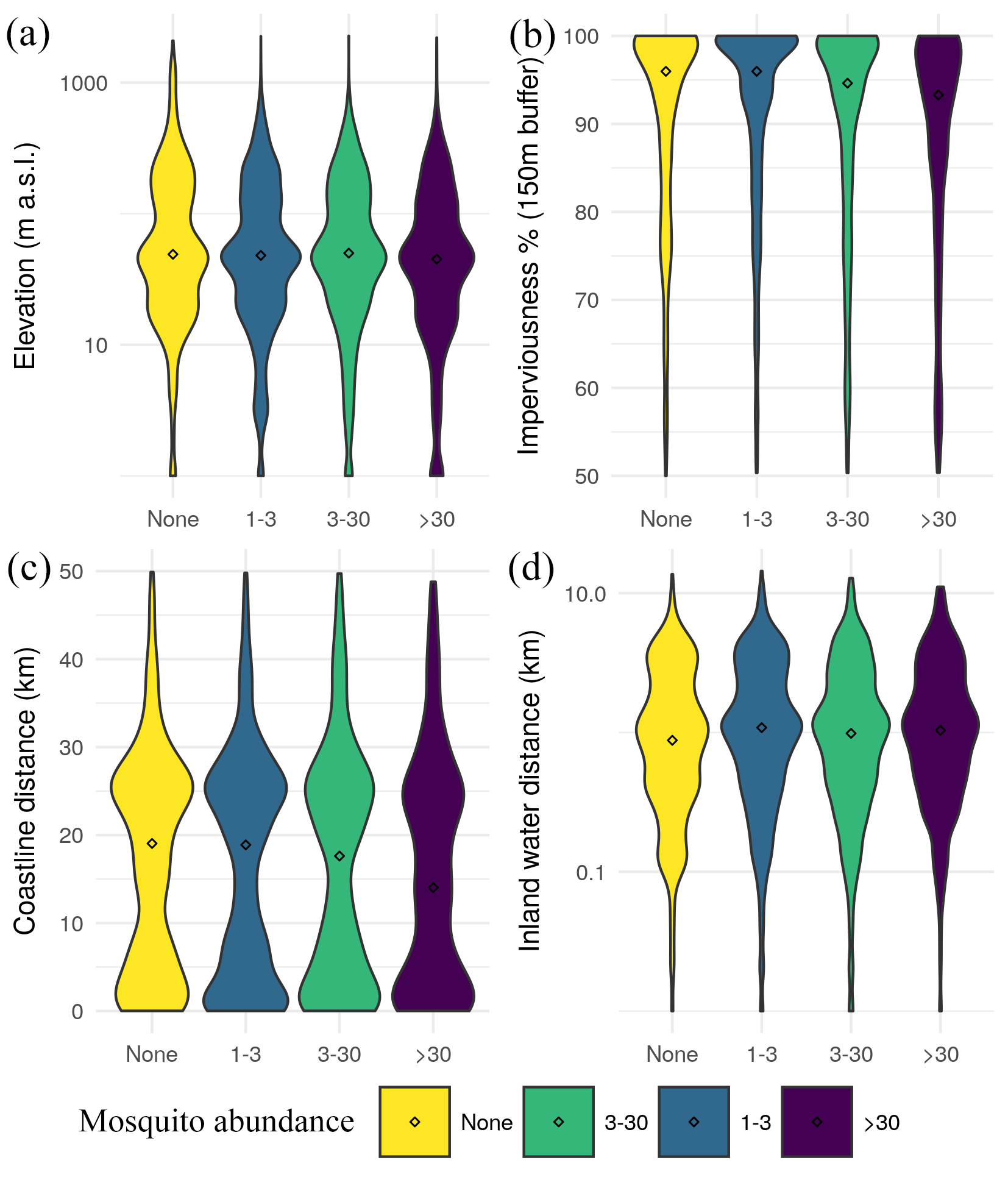

Supplement: Supplementary file 1 [file ijerph-17-07872-s001.zip › supmat/FigureS4.png]

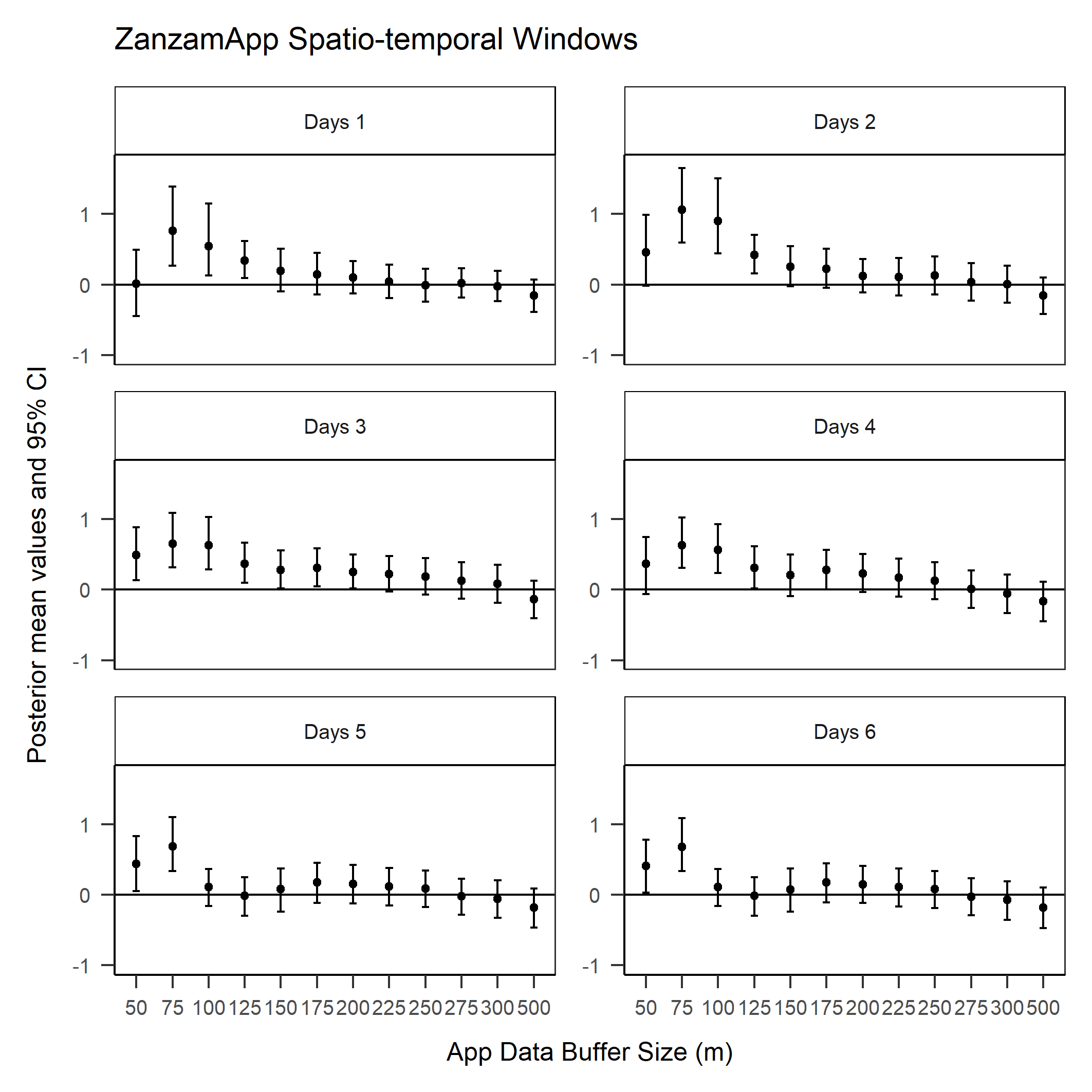

Supplement: Supplementary file 1 [file ijerph-17-07872-s001.zip › supmat/FigureS5.tiff]
